# Interplay of polygenic liability with birth-related, somatic, and psychosocial factors in anorexia nervosa risk: a nationwide study

**DOI:** 10.1017/S0033291724000175

**Published:** 2024-07

**Authors:** Natalie M. Papini, Emily Presseller, Cynthia M. Bulik, Katrine Holde, Janne T. Larsen, Laura M. Thornton, Clara Albiñana, Bjarni J. Vilhjálmsson, Preben B. Mortensen, Zeynep Yilmaz, Liselotte V. Petersen

**Affiliations:** 1 Department of Health Sciences, Northern Arizona University, Flagstaff, AZ, USA; 2 Department of Psychiatry, University of North Carolina at Chapel Hill, Chapel Hill, NC, USA; 3 Department of Psychological and Brain Sciences, Drexel University, Philadelphia, PA, USA; 4 Center for Weight, Eating, and Lifestyle Science, Drexel University, Philadelphia, PA, USA; 5 Department of Nutrition, University of North Carolina at Chapel Hill, Chapel Hill, NC, USA; 6 Department of Medical Epidemiology and Biostatistics, Karolinska Institutet, Stockholm, Sweden; 7 National Centre for Register-based Research, Aarhus BSS, Aarhus University, Aarhus, Denmark; 8 Lundbeck Foundation Initiative for Integrative Psychiatric Research (iPSYCH), Aarhus University, Aarhus, Denmark; 9 Bioinformatic Research Centre, Aarhus University, Aarhus, Denmark; 10 Novo Nordisk Foundation Center for Genomic Mechanisms of Disease, Broad Institute of MIT and Harvard, Cambridge, MA, USA; 11 Department of Biomedicine, Aarhus University, Aarhus, Denmark

**Keywords:** anorexia nervosa, epidemiology, genetics

## Abstract

**Background:**

Although several types of risk factors for anorexia nervosa (AN) have been identified, including birth-related factors, somatic, and psychosocial risk factors, their interplay with genetic susceptibility remains unclear. Genetic and epidemiological interplay in AN risk were examined using data from Danish nationwide registers. AN polygenic risk score (PRS) and risk factor associations, confounding from AN PRS and/or parental psychiatric history on the association between the risk factors and AN risk, and interactions between AN PRS and each level of target risk factor on AN risk were estimated.

**Methods:**

Participants were individuals born in Denmark between 1981 and 2008 including nationwide-representative data from the iPSYCH2015, and Danish AN cases from the Anorexia Nervosa Genetics Initiative and Eating Disorder Genetics Initiative cohorts. A total of 7003 individuals with AN and 45 229 individuals without a registered AN diagnosis were included. We included 22 AN risk factors from Danish registers.

**Results:**

Risk factors showing association with PRS for AN included urbanicity, parental ages, genitourinary tract infection, and parental socioeconomic factors. Risk factors showed the expected association to AN risk, and this association was only slightly attenuated when adjusted for parental history of psychiatric disorders or/and for the AN PRS. The interaction analyses revealed a differential effect of AN PRS according to the level of the following risk factors: sex, maternal age, genitourinary tract infection, C-section, parental socioeconomic factors and psychiatric history.

**Conclusions:**

Our findings provide evidence for interactions between AN PRS and certain risk-factors, illustrating potential diverse risk pathways to AN diagnosis.

## Background

Anorexia nervosa (AN) is a serious eating disorder marked by significantly low body weight accompanied by restrictive eating, weight and/or body shape disturbance, fear of weight gain, and significant impairment in physical, social, and psychological functioning (APA, 2013). The lifetime prevalence of AN in Europe is 1.0 to 4.0% for females and 0.3 to 0.7% for males (Keski-Rahkonen & Mustelin, 2016). AN onset mostly occurs in adolescence, and it is estimated that 10–20% of people report chronic, persistent AN (Keski-Rahkonen & Mustelin, 2016; Speciani et al., 2021; Steinhausen, 2009; Udo & Grilo, 2018). Worldwide, incidence rates of AN have remained stable over the last 30 years, although they have increased among certain subsets of the population (e.g. younger individuals aged <15 years) (van Eeden, van Hoeken, & Hoek, 2021). AN ranks among the psychiatric disorders with strongest association with premature relative mortality, surpassed only by opioid use disorder (Arcelus, Mitchell, Wales, & Nielsen, 2011; Chesney, Goodwin, & Fazel, 2014; Wonderlich, Bulik, Schmidt, Steiger, & Hoek, 2020), with 5–6 times higher mortality rate than the general population (van Eeden et al., 2021; van Hocken & Hoek 2020; Wonderlich et al., 2020).

Family and twin studies of AN provide evidence for notable heritability. For instance, females who have a relative with AN are 11 times more likely to develop AN than those who do not have a relative with AN (Strober, Freeman, Lampert, Diamond, & Kaye, 2000). Twin studies report AN heritability estimates ranging from 0.48 to 0.74 (Yilmaz, Hardaway, & Bulik, 2015). A total of four genome-wide association studies (GWAS) have been conducted examining risk loci for AN (Boraska et al., 2014; Duncan et al., 2017; Wang et al., 2011; Watson et al., 2021), estimating ~11–17% of AN heritability to be attributed to common genetic variants and identifying eight genome-wide significant loci including four single-gene loci (*CADM1*, *MGMT*, *FOXP1*, and *PTBP2*) in AN etiology (Watson et al., 2019b). Recent GWAS efforts with increased sample sizes have resulted in greater gene discovery in the field of complex trait genomics (Watson et al., 2021). With each GWAS variant accounting for a small increase in underlying genetic risk factors for AN, a more powerful way to predict genomic risk is accomplished with polygenic risk scores (PRS: the sum of an individual's risk alleles weighted by effect size; Uffelmann et al., 2021). Many studies to date have used PRS alone to predict complex traits and conditions. When combined with epidemiological risk factors, more comprehensive risk modeling is achieved (Kullo et al., 2022; Lee et al., 2022). Additionally, the relationship among PRS, childhood exposure to nitrogen dioxide, and schizophrenia outcomes were examined in a Danish population cohort, where increased PRS for schizophrenia was positively associated with higher nitrogen dioxide exposure during childhood (Horsdal et al., 2019). These examples highlight the utility of combining PRS with epidemiological risk factors to gain greater insight into the etiology of psychiatric disorders. Similar to other common psychiatric disorders, AN is highly polygenic in nature (where individual genes of small effect contribute to its occurrence), and as such PRS is a promising tool for identifying AN genetic risk (Baker, Schaumberg, & Munn-Chernoff, 2017; Wray et al., 2014), although research evaluating their clinical utility and ethical issues related to their application is needed (Wray et al., 2021).

In addition to genetic factors, epidemiological factors also significantly contribute to the etiology of AN (Yao et al., 2021). Several types of risk factors for AN have been identified, including birth-related factors, early childhood adversities, and psychosocial risk factors. In the present study, we restricted our selection to factors presented in the literature found to be significantly associated with AN risk that were also available in the Danish registers. For example, higher parental educational attainment has been linked to increased risk for AN (Ahrén, Chiesa, af Klinteberg, & Koupil, 2012; Goodman, Heshmati, & Koupil, 2014; Koch et al., 2022; Sundquist, Ohlsson, Winkleby, Sundquist, & Crump, 2016). Additional mixed evidence has been identified regarding the relationship between parental income and risk for AN, with some studies finding a significant positive association (Koch et al., 2022; Lindberg & Hjern, 2003), and others failing to identify an association independent of parental education (Goodman et al., 2014). Maternal genitourinary tract infection, higher maternal and paternal age at birth, multiple births, Cesarean section, congenital malformations of the mouth or digestive system, and preterm birth have been consistently linked to increased risk for AN (Larsen, Bulik, Thornton, Koch, & Petersen, 2021; Lindberg & Hjern, 2003; Marzola et al., 2021). Smoking during pregnancy, birth weights above 4500 g, and birth after 42 weeks of gestation have been associated with significantly lower risk for AN (Ekblad, Gissler, Lehtonen, & Korkeila, 2010; Larsen et al., 2021; Marzola et al., 2021; Watson et al., 2019a). Psychosocial and childhood adversity risk factors that have been identified include parental psychiatric history, intercountry adoption, and foster care before age 13 (Lindberg & Hjern, 2003). We included urbanicity – which is associated with lower AN risk in the case of living in a rural setting in Denmark (Koch et al., 2022; Larsen et al., 2021) – which has been investigated in a Dutch study but was not replicated, possibly due to insufficient sample size (van Son, van Hoeken, Bartelds, van Furth, & Hoek, 2006). We also included childhood infections which – as is also in the case of other psychiatric disorders – are associated with higher AN risk (Breithaupt et al., 2019). Genes and environment have been hypothesized to work together in the etiology of AN through gene-environment correlation and gene-by-environment interaction (Baker et al., 2017). Examination of both epidemiological risk factors and biology – while being mindful that the interaction will likely to be complex in nature, with epidemiological factors also being influenced by genetics – has the potential to facilitate more personalized risk prediction in individuals with AN.

The aim of the present study is to examine interplay between polygenetic liability and risk factors in the risk of AN, utilizing data obtained from Danish national registers. Risk factors examined included birth-related, somatic, and psychosocial factors. To accomplish this, associations, confounding, and interactions were estimated. Associations between AN PRS and each risk factor in the population were evaluated, with significant findings suggesting that AN PRS is associated with (or even affect) an individual's likelihood to be exposed to the risk factor. Next, AN PRS and parental psychiatric history were included as confounding variables when examining the association between the risk factors and AN incidence, with the results revealing to what extent the associations attenuate when adjusted for confounding from AN PRS and parental psychiatric history. Lastly, we determined the interaction between AN PRS and each risk factor on AN incidence, showing whether the association involving AN PRS for AN risk is stronger at one level compared to another level of the risk factors.

For the association analyses, we hypothesized that the AN PRS would be positively associated with the risk factors; i.e., the genetic load of AN risk was hypothesized to be higher for individuals exposed to the risk factor compared with unexposed individuals. Accordingly, part of the increased AN risk in people exposed to the risk factor could be attributable to higher AN genetic load. Consequently, we hypothesized that the increased AN risk associated with the risk factors would be attenuated after adjusting for AN PRS and family history in the confounding analyses. Based on the literature, the factors we hypothesized to have the strongest associations with AN risk were sex, maternal education, and familial psychiatric history, as these factors are known to be affected by genetics, and could, speculatively, show interactions with the PRS on AN risk. Potential interpretations could be that genetic load may play a stronger role at one level of a given risk factor than other levels of the risk factor, which could point to diverse pathways toward an AN diagnoses.

## Methods

### Sample

We used data from the iPSYCH consortium and its established population-based case-cohort sample (iPSYCH2015; Bybjerg-Grauholm et al., 2020), and the Danish arm of the Anorexia Nervosa Genetics Initiative (ANGI; Thornton et al., 2018) and Eating Disorders Genetics Initiative (EDGI; Bulik et al., 2021b) cohorts, all of which are described in detail elsewhere. The samples were drawn from individuals born in Denmark between 1 May 1981 and 31 December 2008 and alive and living in Denmark on their first birthday (*N* = 1 768 952 in the background population). Genetic information was based in DNA extracted from neonatal dried blood spot samples kept in the Danish Newborn Screening Biobank. Retrieval of blood samples, genotyping, and quality control have been described elsewhere (Pedersen et al., 2018). The analytic cohort included 45 458 randomly selected individuals and 7003 individuals with an AN diagnosis (ICD-10 F50.0 and F50.1) after age 1 in the Danish Psychiatric Central Research Register (DPCRR) or the Danish National Patient Register (DNPR), an electronic register that contains data on all psychiatric hospital admissions from 1969 to present as well as outpatient admissions since 1995 (Lynge, Sandegaard, & Rebolj, 2011; Mors, Perto, & Mortensen, 2011; Munk-Jorgensen et al., 1997). The case-cohort design has some advantages to case-control studies: the randomly selected cohort allows estimation of relative risk rather than odds ratios, and the cohort can be reused for comparison to different case groups. Presently, it is used in iPSYCH and for the AN case group. Out of the 45 458 individuals randomly selected, there were 229 who were registered with an AN diagnosis.

### Risk factors

We examined several established risk factors available in the Danish registers, such as birth-related, somatic, and psychosocial factors. Demographic factors including sex, urbanicity at birth, and parental age at birth were extracted from The Danish Civil Registration System. Information on birth-related risk factors, including gestational age, birth weight, and method of delivery were extracted from the Danish Medical Birth Register (DMBR; Bliddal, Broe, Pottegård, Olsen, & Langhoff-Roos, 2018; Pedersen, 2011). The DNPR was used to identify other birth-related and somatic factors, including maternal infections during pregnancy, and congenital malformations of the mouth and digestive system, and childhood infections (Lynge et al., 2011). Maternal genitourinary infection during pregnancy, and any infections during pregnancy (including genitourinary infection) were both analyzed. Data on psychosocial factors such as out-of-home and in-home care were found in the Register of Support for Children and Adolescents. Data on other psychosocial risk factors, such as parental income and education were extracted from Denmark's socioeconomic registers (Petersson, Baadsgaard, & Thygesen, 2011). Finally, the DPCRR was used to examine parental psychiatric history (Mors et al., 2011), the variables were categorized into parental eating disorders, other psychiatric diagnoses in mother or the father, and no psychiatric disorders in the parents.

### Polygenic risk score calculation

AN PRS was calculated using LDpred2 (Privé, Arbel, & Vilhjálmsson, 2020) using summary statistics from the Psychiatric Genomics Consortium Eating Disorders Working Group (PGC-ED) Freeze 2 AN GWAS (Watson et al., 2019b) after the removal of Danish AN cases and controls (discovery sample for PRS: 12 400 AN cases and 35 046 controls). After being standardized in the random sample, this comprised the application of AN PRS to estimate associations, confounding, and interactions with each risk factor in the target Danish sample.

The study was approved by the Danish Scientific Ethics Committee, the Danish Health Data Authority, the Danish Data Protection Agency, and Danish Newborn Screening Biobank Steering. The Danish Scientific Ethics Committee, in accordance with Danish legislation, has, for this study, waived the need for informed consent in biomedical research based on existing biobanks (Mortensen, 2019; Pedersen et al., 2018).

### Statistical analyses

For the association analyses we included only the randomly selected participant. We used multinomial logistic regression with the included risk factors as outcomes and the PRS for AN as the exposure to estimate the association between the levels of the risk factors and the PRS. For the confounding and interaction effect case-cohort analyses were conducted using weighted Cox models, where the individuals were followed from age 6 until AN diagnosis or until death/emigration or 31 December 2016, whichever occurred first. The weights represented the inverse probability of a participant being selected; for the random part of the sample (size of main study population (1 768 952) divided by the number without AN selected, here 45 229), and for AN cases the weight was equal to 1, as all AN cases from the main study population were included. With these models, we investigated potential confounding from the PRS and parental psychiatric history for each risk factor for AN, unadjusted (model 1), adjusting for PRS (model 2), parental psychiatric history (model 3), and both (model 4) by including these variables as covariates in the model. Furthermore, we looked at potential interactions allowing differential linear effects of PRS across levels of the risk factors by including an interaction term between the PRS and the risk factor in the weighted Cox model. In all analyses we adjusted for the first 10 genetic principal components. For the confounding analyses, model 1 has specific hypotheses for each risk factor. The confounding analyses (model 2–4) were not tested by specific tests; instead the adjusted estimates are shown. For the association and interaction analyses we regard as multiple testing situations, accordingly in these analyses, we also included an adjusted *p* value using the Benjamini–Hochberg correction (adjustment was conducted for all analyses within each table). Missing values were handled by complete case analyses, as they were rare (see Table 1). We completed all analyses and generated plots using R version 4.0.4 and R Studio version 1.4.1106.

**Table 1.** Descriptive of cases and controls in our sample

	Cases	Controls
Total, *N* (%)	7003 (100%)	45 229 (100%)
Female, *N* (%)	6518 (93.1%)	22 117 (48.9%)
Age in 2016, Median (5–95% centiles) years	24.4 (15.5–34.1)	21.9 (11.0–33.9)
European ancestry, *N* (%)	6569 (93.8%)	40 193 (88.9%)
High maternal education, *N* (%)	2507 (35.8%)	14 163 (31.3%)

## Results

The full study population included 45 458 population-based cohort members (23 129 [50.9%] males; 22 329 [49.1%] females), plus the 7003 cases of AN (*n* = 485 [6.9%] males; *n* = 6518 [93.1%] females). The mean age at first AN diagnosis was 17.6 years in females and 16.0 years in males.

### PRS and risk factor associations

**Figure 1.**
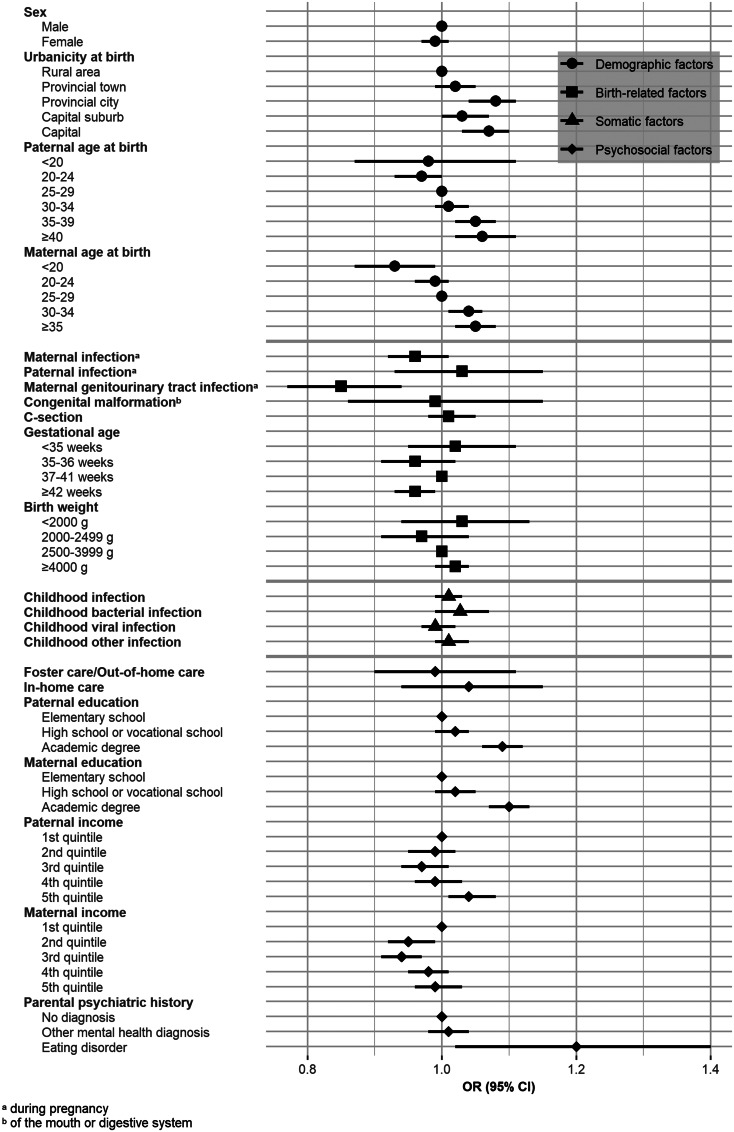
Association analysis: odds ratio per 1 standard deviation increase in anorexia nervosa polygenic risk score across levels of risk factors for anorexia nervosa.

Our analysis revealed that AN PRS was significantly positively associated with urbanicity, paternal age at birth, maternal age at birth, maternal genitourinary tract infection during pregnancy, paternal and maternal education, and both paternal and maternal income in the random sample of our study (excluding those only in the sample of individuals with AN) (see Fig. 1). AN PRS was not significantly associated with sex, paternal or maternal infection during pregnancy, congenital malformation of the mouth or digestive system, method of delivery, gestational age, birth weight, childhood infection, foster care/out-of-home care, in-home care, or parental psychiatric history (see Fig. 1 and online Supplementary Table S1).

### Known AN risk factors and confounding analyses

Hazard ratios for AN were significantly higher among females and individuals who live in urban areas (capital cities, capital suburbs, provincial cities, and provincial towns) compared with rural areas (see Table 2). Parental age was also significantly associated with hazard ratios for AN, with paternal age > 30 years old and maternal age between 30–34 years old associated with increased risk for AN (compared with paternal and maternal ages 25–29), further, hazard ratios for AN were significantly lower among individuals born to a father 20–24 years old at birth (compared to 25–29 years old), and individuals born to a mother < 20 or 20–24 years old at birth (compared to 25–29 years old).

**Table 2.** Confounding analysis: hazard ratios of genetic and environmental risk factors for anorexia nervosa

	*N* cases	HR_1_	95% CI	HR_2_^a^	95% CI	HR_3_^b^	95% CI	HR_4_^c^	95% CI
	*N* = 7003 AN cases								
*Demographic factors*									
Sex									
Male	485 (6.9%)	1 (ref)		1 (ref)		1 (ref)		1 (ref)	
Female	6518 (93.1%)	14.00	(12.74–15.39)	14.03	(12.76–15.43)	13.98	(12.72–15.37)	14.00	(12.73–15.40)
Urbanicity									
Rural area	2133 (30.5%)	1 (ref)		1 (ref)		1 (ref)		1 (ref)	
Provincial town	1811 (25.9%)	1.08	(1.01–1.15)	1.07	(1.00–1.15)	1.07	(1.00–1.15)	1.07	(1.00–1.14)
Provincial city	1012 (14.5%)	1.42	(1.31–1.54)	1.39	(1.27–1.51)	1.42	(1.30–1.54)	1.38	(1.27–1.51)
Capital suburb	1019 (14.6%)	1.25	(1.15–1.36)	1.23	(1.13–1.34)	1.25	(1.15–1.36)	1.23	(1.13–1.34)
Capital	1028 (14.7%)	1.48	(1.36–1.61)	1.44	(1.33–1.57)	1.47	(1.35–1.60)	1.44	(1.32–1.57)
Paternal age at birth									
<20	36 (0.5%)	0.72	(0.50–1.02)	0.73	(0.51–1.04)	0.70	(0.49–1.00)	0.72	(0.50–1.02)
20–24	585 (8.4%)	0.81	(0.73–0.89)	0.82	(0.74–0.91)	0.80	(0.72–0.88)	0.81	(0.73–0.89)
25–29	2201 (31.4%)	1 (ref)		1 (ref)		1 (ref)		1 (ref)	
30–34	2397 (34.2%)	1.12	(1.06–1.20)	1.12	(1.05–1.20)	1.13	(1.06–1.20)	1.13	(1.06–1.20)
35–39	1208 (17.2%)	1.15	(1.07–1.25)	1.14	(1.05–1.23)	1.15	(1.07–1.24)	1.13	(1.05–1.22)
⩾40	576 (8.2%)	1.24	(1.12–1.37)	1.22	(1.10–1.35)	1.23	(1.11–1.36)	1.21	(1.09–1.34)
Maternal age at birth									
<20	113 (1.6%)	0.62	(0.51–0.76)	0.65	(0.53–0.79)	0.59	(0.49–0.73)	0.62	(0.51–0.76)
20–24	1191 (17%)	0.77	(0.71–0.83)	0.77	(0.72–0.83)	0.76	(0.71–0.82)	0.76	(0.71–0.82)
25–29	2813 (40.2%)	1 (ref)		1 (ref)		1 (ref)		1 (ref)	
30–34	2122 (30.3%)	1.13	(1.06–1.20)	1.12	(1.05–1.19)	1.13	(1.06–1.21)	1.12	(1.05–1.20)
⩾35	764 (10.9%)	1.08	(0.98–1.17)	1.06	(0.97–1.16)	1.07	(0.98–1.16)	1.05	(0.96–1.15)
*Birth-related factors*									
Maternal infection during pregnancy									
No	6740 (96.2%)	1 (ref)		1 (ref)		1 (ref)		1 (ref)	
Yes	263 (3.8%)	1.10	(0.96–1.27)	1.10	(0.96–1.27)	1.08	(0.94–1.24)	1.08	(0.95–1.25)
Paternal infection during pregnancy									
No	6962 (99.4%)	1 (ref)		1 (ref)		1 (ref)		1 (ref)	
Yes	41 (0.6%)	0.84	(0.6–1.17)	0.84	(0.6–1.17)	0.83	(0.59–1.15)	0.82	(0.59–1.15)
Maternal genitourinary tract infection during pregnancy									
No	6943 (99.1%)	1 (ref)		1 (ref)		1 (ref)		1 (ref)	
Yes	60 (0.9%)	1.97	(1.49–2.62)	2.09	(1.57–2.78)	1.88	(1.42–2.5)	2.00	(1.5–2.66)
Congenital malformation of the mouth or digestive system									
No	6983 (99.7%)	1 (ref)		1 (ref)		1 (ref)		1 (ref)	
Yes	20 (0.3%)	1.07	(0.67–1.72)	1.09	(0.68–1.75)	1.02	(0.64–1.64)	1.05	(0.65–1.69)
C-section									
No	6395 (91.3%)	1 (ref)		1 (ref)		1 (ref)		1 (ref)	
Yes	608 (8.7%)	1.21	(1.11–1.33)	1.21	(1.1–1.32)	1.20	(1.1–1.32)	1.20	(1.09–1.31)
Gestational age									
<35 weeks	132 (1.9%)	1.32	(1.09–1.61)	1.31	(1.08–1.60)	1.30	(1.07–1.58)	1.29	(1.06–1.57)
35–36 weeks	188 (2.7%)	0.97	(0.83–1.14)	1.00	(0.85–1.17)	0.96	(0.81–1.12)	0.98	(0.84–1.15)
37–41 weeks	6111 (87.3%)	1 (ref)		1 (ref)		1 (ref)		1 (ref)	
⩾42 weeks	572 (8.2%)	0.85	(0.77–0.93)	0.86	(0.78–0.94)	0.85	(0.77–0.93)	0.86	(0.78–0.95)
Birth weight									
<2000 g	92 (1.3%)	1.19	(0.95–1.5)	1.18	(0.93–1.49)	1.17	(0.93–1.47)	1.15	(0.91–1.45)
2000–2499 g	191 (2.7%)	1.07	(0.91–1.26)	1.10	(0.93–1.29)	1.05	(0.9–1.23)	1.08	(0.92–1.27)
2500–3999 g	5699 (81.4%)	1 (ref)		1 (ref)		1 (ref)		1 (ref)	
⩾4000 g	989 (14.1%)	0.80	(0.75–0.87)	0.80	(0.75–0.87)	0.81	(0.75–0.87)	0.81	(0.75–0.87)
Missing	32 (0.5%)								
*Somatic factors*									
Childhood infection									
No	5430 (77.5%)	1 (ref)		1 (ref)		1 (ref)		1 (ref)	
Yes	1573 (22.5%)	1.00	(0.94–1.06)	0.99	(0.93–1.06)	0.99	(0.93–1.05)	0.98	(0.92–1.05)
Childhood bacterial infection									
No	6570 (93.8%)	1 (ref)		1 (ref)		1 (ref)		1 (ref)	
Yes	433 (6.2%)	1.15	(1.04–1.28)	1.15	(1.03–1.28)	1.14	(1.03–1.27)	1.14	(1.02–1.27)
Childhood viral infection									
No	6235 (89%)	1 (ref)		1 (ref)		1 (ref)		1 (ref)	
Yes	768 (11%)	1.07	(0.99–1.16)	1.07	(0.99–1.17)	1.06	(0.98–1.15)	1.06	(0.98–1.16)
Childhood other infection									
No	6105 (87.2%)	1 (ref)		1 (ref)		1 (ref)		1 (ref)	
Yes	898 (12.8%)	0.96	(0.89–1.03)	0.95	(0.88–1.03)	0.95	(0.88–1.02)	0.94	(0.87–1.02)
Psychosocial factors									
Foster care/Out-of-home care									
No	6951 (99.3%)	1 (ref)		1 (ref)		1 (ref)		1 (ref)	
Yes	52 (0.7%)	0.77	(0.57–1.03)	0.77	(0.57–1.03)	0.64	(0.47–0.86)	0.64	(0.48–0.87)
In-home care									
No	6952 (99.3%)	1 (ref)		1 (ref)		1 (ref)		1 (ref)	
Yes	51 (0.7%)	1.00	(0.74–1.35)	0.98	(0.73–1.33)	0.88	(0.65–1.19)	0.87	(0.64–1.18)
Paternal education									
Elementary school	1343 (19.2%)	1 (ref)		1 (ref)		1 (ref)		1 (ref)	
High school or vocational school	3208 (45.8%)	1.18	(1.11–1.27)	1.18	(1.1–1.27)	1.21	(1.13–1.3)	1.21	(1.12–1.3)
Academic degree	2210 (31.6%)	1.60	(1.49–1.73)	1.56	(1.45–1.68)	1.65	(1.53–1.78)	1.60	(1.49–1.73)
Missing	242 (3.5%)								
Maternal education									
Elementary school	1610 (23%)	1 (ref)		1 (ref)		1 (ref)		1 (ref)	
High school or vocational school	2762 (39.4%)	1.26	(1.18–1.35)	1.25	(1.17–1.34)	1.30	(1.21–1.39)	1.28	(1.2–1.37)
Academic degree	2507 (35.8%)	1.66	(1.55–1.78)	1.60	(1.5–1.72)	1.71	(1.6–1.83)	1.65	(1.54–1.77)
Missing	124 (1.8%)								
Paternal income									
1st quintile	797 (11.4%)	1 (ref)		1 (ref)		1 (ref)		1 (ref)	
2nd quintile	1126 (16.1%)	1.00	(0.91–1.11)	1.01	(0.91–1.11)	1.02	(0.92–1.12)	1.02	(0.92–1.13)
3rd quintile	1445 (20.6%)	1.13	(1.03–1.25)	1.14	(1.04–1.26)	1.17	(1.06–1.29)	1.18	(1.07–1.3)
4th quintile	1556 (22.2%)	1.16	(1.05–1.27)	1.16	(1.05–1.27)	1.20	(1.09–1.32)	1.20	(1.09–1.32)
5th quintile	2022 (28.9%)	1.40	(1.27–1.53)	1.37	(1.25–1.5)	1.45	(1.32–1.59)	1.42	(1.29–1.56)
Missing	57 (0.8%)								
Maternal income									
1st quintile	892 (12.7%)	1 (ref)		1 (ref)		1 (ref)		1 (ref)	
2nd quintile	1356 (19.4%)	0.99	(0.9–1.08)	1.00	(0.91–1.1)	1.00	(0.91–1.09)	1.01	(0.92–1.11)
3rd quintile	1488 (21.2%)	1.00	(0.91–1.1)	1.02	(0.93–1.12)	1.02	(0.93–1.12)	1.04	(0.95–1.14)
4th quintile	1545 (22.1%)	1.10	(1–1.2)	1.11	(1.01–1.21)	1.12	(1.02–1.23)	1.13	(1.03–1.24)
5th quintile	1701 (24.3%)	1.26	(1.16–1.38)	1.27	(1.16–1.39)	1.30	(1.19–1.43)	1.31	(1.19–1.43)
Missing	21 (0.3%)								
Parental psychiatric history									
No diagnosis	6101(87.1%)	1 (ref)		1 (ref)		–		–	
Other psychiatric diagnosis	854 (12.2%)	1.25	(1.16–1.36)	1.24	(1.14–1.34)	–		–	
Eating disorder diagnosis	48 (0.7%)	3.85	(2.69–5.52)	3.62	(2.50–5.24)	–		–	

*Abbreviations: N*, sample size; HR, hazard ratio; CI, confidence interval.

a HR_1_ with additional adjustment for anorexia nervosa polygenic risk score.

b HR_1_ with additional adjustment for parental psychiatric history.

c HR_1_ with additional adjustment for anorexia nervosa polygenic risk score and parental psychiatric history.

The risk of AN was higher among individuals with mothers who experienced genitourinary tract infection during pregnancy (compared with mothers who did not), and individuals born via C-section (compared with vaginal birth). AN risk was also higher among individuals with gestational age < 35 weeks (compared to 37–41 weeks) and lower among individuals with gestational age ⩾ 42 weeks (compared to 37–41 weeks). Similarly, AN risk was inversely associated with birth weight, and significantly decreased for individuals with birth weight ⩾ 4000 g (compared to 2500–3999 g).

Individuals born to a father and/or mother with more than an elementary school education (compared with elementary school education only), individuals with fathers whose income was in the upper three quintiles (compared with the lowest quintile), and individuals with mothers whose income was in the highest quintile (compared with the lowest quintile) demonstrated elevated hazard ratios for AN. Finally, both general parental psychiatric history and parental history of eating disorders were associated with significantly elevated AN hazard ratios, compared with no parental psychiatric history.

To evaluate confounding, all the above risk factors were adjusted for AN PRS (model 2), parental psychiatric history (model 3), and both AN PRS and parental psychiatric history (model 4). All adjustments resulted in only minor changes to the associations between risk factors and AN.

### Interaction analyses

Table 3 summarizes the Cox models including interaction terms between AN PRS and risk factors on AN risk. Significant interaction effects were identified for sex, maternal age at birth, maternal genitourinary tract infection, method of delivery, paternal education, maternal education, paternal income, and parental psychiatric history. Specifically, higher AN PRS had a larger effect on risk for AN diagnosis among females, individuals born to mothers younger than 20 and older than 25 years old, individuals born through cesarean section, individuals born when mother experienced genitourinary tract infection during pregnancy, individuals born to fathers or mothers with an academic degree, and individuals with fathers whose income was in the highest quintile.

**Table 3.** Interaction analysis: differential (linear) effect of anorexia nervosa polygenic risk score across levels of risk factors for anorexia nervosa

	HR per s.d. of AN PRS	95% CI	*p* value	Adj. *p* value
*Demographic factors*				
Sex				
Male	1.12	(1.09–1.14)		
Female	1.87	(1.78–1.95)	<0.001	<0.001**
Urbanicity				
Rural area	1.45	(1.38–1.52)		
Provincial city	1.59	(1.47–1.71)		
Provincial town	1.43	(1.36–1.50)		
Capital suburb	1.44	(1.34–1.55)		
Capital	1.52	(1.41–1.65)	0.16	0.31
Paternal age at birth				
<20	1.36	(1.04–1.79)		
20–24	1.38	(1.27–1.49)		
25–29	1.44	(1.37–1.50)		
30–34	1.51	(1.44–1.58)		
35–39	1.50	(1.40–1.60)		
⩾40	1.52	(1.38–1.67)	0.35	0.55
Maternal age at birth				
<20	1.56	(1.33–1.83)		
20–24	1.33	(1.26–1.40)		
30–34	1.53	(1.45–1.62)		
25–29	1.50	(1.43–1.56)		
⩾35	1.48	(1.36–1.61)	<0.05	<0.05**
*Birth-related factors*				
Maternal infection during pregnancy				
No	1.47	(1.43–1.51)		
Yes	1.49	(1.3–1.71)	0.79	0.91
Paternal infection during pregnancy				
No	1.47	(1.43–1.51)		
Yes	1.54	(1.15–2.07)	0.75	0.91
Maternal genitourinary tract infection during pregnancy				
No	1.46	(1.42–1.5)		
Yes	3.59	(2.54–5.08)	<0.001	<0.001**
Congenital malformation of the mouth or digestive system				
No	1.47	(1.43–1.51)		
Yes	1.20	(0.56–2.60)	0.61	0.82
C-section				
No	1.45	(1.41–1.49)		
Yes	1.67	(1.53–1.83)	<0.05	<0.05*
Gestational age				
<35 weeks	1.56	(1.23–1.97)		
35–36 weeks	1.77	(1.52–2.07)		
37–41 weeks	1.46	(1.42–1.51)		
⩾42 weeks	1.44	(1.33–1.57)	0.10	0.25
Birth weight				
<2000 g	1.47	(1.13–1.91)		
2000–2499 g	1.55	(1.30–1.83)		
2500–3999 g	1.47	(1.42–1.52)		
⩾4000 g	1.44	(1.35–1.53)	0.87	0.91
*Somatic factors*				
Childhood infection				
No	1.48	(1.44–1.53)		
Yes	1.42	(1.35–1.50)	0.21	0.38
Childhood bacterial infection				
No	1.47	(1.43–1.51)		
Yes	1.48	(1.32–1.66)	0.85	0.91
Childhood viral infection				
No	1.47	(1.43–1.51)		
Yes	1.46	(1.34–1.59)	0.87	0.91
Childhood other infection				
No	1.48	(1.44–1.53)		
Yes	1.40	(1.30–1.50)	0.13	0.28
*Psychosocial factors*				
Foster care/Out-of-home care				
No	1.47	(1.43–1.51)		
Yes	1.31	(1.04–1.66)	0.36	0.55
In-home care				
No	1.47	(1.43–1.51)		
Yes	1.35	(1.00–1.82)	0.57	0.82
Paternal education				
Elementary school	1.32	(1.25–1.40)		
High school or vocational school	1.46	(1.41–1.52)		
Academic degree	1.65	(1.56–1.74)	<0.001	<0.001**
Maternal education				
Elementary school	1.36	(1.30–1.42)		
High school or vocational school	1.46	(1.40–1.52)		
Academic degree	1.60	(1.52–1.68)	<0.001	<0.001**
Paternal income				
1st quintile	1.40	(1.31–1.51)		
2nd quintile	1.34	(1.27–1.42)		
3rd quintile	1.46	(1.38–1.56)		
4th quintile	1.53	(1.44–1.62)		
5th quintile	1.55	(1.47–1.64)	<0.05	<0.05*
Maternal income				
1st quintile	1.38	(1.29–1.48)		
2nd quintile	1.46	(1.38–1.55)		
3rd quintile	1.53	(1.45–1.62)		
4th quintile	1.44	(1.35–1.52)		
5th quintile	1.50	(1.41–1.60)	0.13	0.28
Parental psychiatric history				
No diagnosis	1.46	(1.41–1.50)		
Other psychiatric disorders	1.52	(1.40–1.65)		
Eating disorder diagnosis	3.08	(1.95–4.85)	<0.05	<0.05*

*Abbreviations:* HR, hazard ratio; CI, confidence interval. *p* values were adjusted using the Benjamini–Hochberg correction table-wise within the association analyses.

*Note.* Interaction using differential linear effect of anorexia nervosa polygenic risk score across the levels of the risk factors. * = significant at the *p* < 0.05 level. ** = significant at the *p* < 0.001 level.

## Discussion

The present study investigated three types of interplay (association, confounding, and interaction) in AN using AN PRS and birth-related, somatic, and psychosocial risk factors. AN PRS was significantly associated with eight risk factors, including urbanicity, paternal and maternal age at birth, maternal genitourinary tract infection during pregnancy, paternal and maternal education, and paternal and maternal income. Despite these associations, AN PRS and parental psychiatric history had minimal confounding effects on the association between the risk factors investigated and a diagnosis of AN. In other words, polygenetic liability and parental psychiatric history may have little influence as confounders of the association between the investigated factors and AN risk. Previously published findings were confirmed (HR_1_ in Table 2) (Breithaupt et al., 2019; Koch et al., 2022; Larsen et al., 2017, 2021; Marzola et al., 2021; Yao et al., 2021). The interaction results provided preliminary evidence for PRS interactions with the risk factors: sex, maternal age at birth, maternal genitourinary tract infection during pregnancy, method of delivery, paternal and maternal education, paternal income, and parental psychiatric history.

This study was the first to examine the association between genetic underpinnings of AN and a series of risk factors in a random sample of 45 458 participants from the general population. We identified that in the general population, higher AN PRS was associated with several AN risk factors, offering preliminary evidence that associations exist between AN PRS and birth-related, somatic, and psychosocial risk factors. In particular, associations between AN PRS and urbanicity, paternal and maternal age at birth, maternal genitourinary tract infection during pregnancy, paternal and maternal education, and paternal and maternal income may suggest that these factors could be impacted by the same genetic mechanisms as AN. It is also plausible that there are environmental or lifestyle factors that influence both the development of AN and these other factors. Further, the risk of AN may be more pronounced in specific environments. For example, growing up in an urban setting or experiencing maternal genitourinary tract infection during pregnancy may increase the risk of AN, either directly or indirectly through other factors such as stress (Phillipou, Gurvich, Castle, & Rossell, 2022) or inflammation (Dalton et al., 2018).

Our finding on the association between higher SES and increased AN risk could be an artifact of treatment seeking behavior. Though Denmark has a universal health care system and the diagnoses and admissions captured by the national registers are publicly funded, a greater awareness of treatment availability among those with higher SES cannot be excluded. However, this pattern is seen only among AN patients: other psychiatric disorders show the opposite pattern in Denmark, and other eating disorders show a pattern somewhere in the middle (Koch et al., 2022).

Our finding of only minor confounding effect from genetic liability to AN risk and parental history of psychiatric disorders suggest these factors act independently from the epidemiological risk factors accessed; however, future iterations of the AN PRS based on larger samples may change this.

Our findings suggest interactions between AN PRS and sex, maternal age at birth, maternal genitourinary infection during pregnancy, method of delivery, paternal and maternal education, paternal income, and parental psychiatric history of an eating disorder, identifying subgroups affected to a higher degree by their genetic liability to AN than others. Maternal antibodies in relation to *in utero* immune-related exposures have been demonstrated as potential risk factors for a number of psychiatric disorders (Braunschweig & Van de Water, 2012; Hall, Willis, Rodriguez, & Schwarz, 2023), including AN (Favaro et al., 2011; Lydholm et al., 2019; Solmi et al., 2020). Furthermore, maternal antibiotic use during pregnancy could also impact neonatal gut microbiome (Dierikx et al., 2020; Qu, Liu, & Miao, 2021), which has also been implicated in the development and maintenance of AN (Bulik, Carroll, & Mehler, 2021a). For psychosocial risk factors, these interactions may be due to the presence of personality features associated with the development of AN and high educational/income attainment, such as perfectionism (Cassin & von Ranson, 2005; Madigan, 2019), autonomy (i.e. independence, control, and need for achievement; Cassin & von Ranson, 2005; de la Fuente, Malpica-Chavarria, Garzon-Umerenkova, & Pachon-Basallo, 2021; Shi & Qu, 2021), neuroticism/anxiety (Kienngam et al. 2022; Marzola, Porliod, Panero, De-Bacco, & Abbate-Daga, 2020; Tucker-Drob, Briley, Engelhardt, Mann, & Harden, 2016), and harm avoidance (Cassin & von Ranson, 2005). The limited number of interactions observed in the present study should be interpreted in light of the challenges of using PRS to examine interactions: namely, limited statistical power and the potential that phenotypic PRS may not necessarily capture genetic variations associated with differential exposure to risk factors (Zhang & Belsky, 2022).

The sizes of the HRs for the risk factors and AN risk were moderate, with only female sex and family eating disorder history standing out with larger relative risks. The association between AN PRS is overall 1.48 per standard deviation, and compared to epidemiological risk factors this is not negligible, though much of the variation AN risk has yet to be explained thus far. From the interaction analyses, the variation of AN PRS association with AN risk across risk factor levels can be considered moderate, with the steady increase of association between AN PRS from low education/income to higher levels of education and income standing out among our other findings.

### Strengths

The present study has several strengths. This study is the first to examine the interplay between polygenic liability of AN and a broad spectrum of birth-related, somatic, and psychosocial risk factors for AN. The sample was large and representative of the Danish population. The use of Danish registers for measurement of AN diagnosis and risk factors allows for comprehensive detection of these factors within the sample.

### Limitations

Present findings must also be considered in light of study limitations. First, we were limited to risk factors available in the Danish registers; accordingly, future studies should examine other factors that have been shown to show prospective associations with AN risk in past research (such as sociocultural factors (Weissman, 2019), involvement in competitive sports (Krentz & Warschburger, 2013; Stoyel, Slee, Meyer, & Serpell, 2020), and premorbid low body mass index (Yilmaz, Gottfredson, Zerwas, Bulik, & Micali, 2019).). Second, AN diagnoses were determined using ICD-10 F50.0 and F50.1 within individuals seeking eating disorder treatment; individuals with AN who did not seek treatment were not captured. The robustness of the PRS across sexes may potentially weaken our ability to reveal interactions since most of the cases and controls in the AN GWAS were female. As a result of this, we are unable to rule out the possibility that the finding may be different in men, which our present Danish sample does not provide the opportunity to explore more closely. Further, we were unable to examine differences in the interplay of risk factors between different subtypes of AN (restricting type and binge eating/purging type). Given the phenotypic differences observed across AN subtypes, it is plausible that specific risk factors could differentiate these subtypes. Although no differences were observed in the polygenic architecture of AN with binge eating v. AN without binge eating (Watson et al., 2019b), future research should investigate differences in the interplay between AN polygenic liability with risk factors relative to the specific subtypes of AN. Despite use of a national registry to provide population estimates, another limitation of this study includes generalizability outside of Denmark, notably for more genetically diverse populations than Danish, which is predominantly European ancestry.

Finally, while we had > 7000 AN cases in our study population and used the largest AN GWAS published to date for calculating AN PRS, it is likely that statistical power may have been limited for detecting interactions of moderate effect sizes.

## Conclusion

The present study elucidates significant interplay of AN polygenic liability genetic and epidemiological risk factors in AN risk. Findings provide evidence for interactions between AN PRS and certain risk factors, illustrating potential diverse AN risk pathways. Future work should replicate this study using populations with more admixed ancestry to determine differences across ancestry groups.

## Supporting information

Papini et al. supplementary material Papini et al. supplementary material

## Supplementary material

The supplementary material for this article can be found at https://doi.org/10.1017/S0033291724000175
