# Supplementary material for: Interplay of polygenic liability with birth-related, somatic, and psychosocial factors in anorexia nervosa risk: a nationwide study
Source: Psychol Med. 2024 Feb 13;54(9):2073–86. doi: 10.1017/S0033291724000175 (PMC11323254; doi:10.1017/S0033291724000175)
Supplement: Papini et al. supplementary material [file S0033291724000175sup001.docx]

*Table S1: Association analysis. Odds Ratio for a 1 standard deviation increase in Anorexia Nervosa Polygenic Risk Score Across Levels of Risk Factors for Anorexia Nervosa.*

|  | **N in random sample** | **OR** | **95 % CI** | **p-value** | **Adj. p-value** |
| --- | --- | --- | --- | --- | --- |
|  | N = 45,458 |  |  |  |  |
| **Demographic factors** | |  |  |  |  |
| **Sex** |  |  |  |  |  |
| Male | 23,129 (50.9%) | 1 (ref) |  |  |  |
| Female | 22,329 (49.1%) | 0.99 | (0.92, 1.01) | 0.38 | 0.52 |
| **Urbanisity at birth** |  |  |  |  |  |
| Rural area | 15,092 (33.2%) | 1 (ref) |  |  |  |
| Provincial town | 12,556 (27.6%) | 1.02 | (1.00, 1.05) |  |  |
| Provincial city | 5,581 (12.3%) | 1.08 | (1.04, 1.11) |  |  |
| Capital suburb | 6,209 (13.7%) | 1.03 | (1.00, 1.07) |  |  |
| Capital | 5,951 (13.1%) | 1.07 | (1.03, 1.10) | <0.001 | **<0.001**** |
| Missing | 69 (0.2%) |  |  |  |  |
| **Paternal age at birth** |  |  |  |  |  |
| 25-29 | 13,883 (30.5%) | 1 (ref) |  |  |  |
| <20 | 296 (0.7%) | 0.983 | (0.871, 1.109) |  |  |
| 20-24 | 4,220 (9.3%) | 0.967 | (0.932, 1.003) |  |  |
| 30-34 | 15,327 (33.7%) | 1.014 | (0.99, 1.038) |  |  |
| 35-39 | 8,070 (17.8%) | 1.045 | (1.015, 1.076) |  |  |
| ≥40 | 3,662 (8.1%) | 1.063 | (1.023, 1.105) | <0.001 | **<0.001**** |
| **Maternal age at birth** |  |  |  |  |  |
| <20 | 1,018 (2.2%) | 0.93 | (0.87, 0.99) |  |  |
| 20-24 | 8,461 (18.6%) | 0.99 | (0.96, 1.01) |  |  |
| 25-29 | 17,300 (38.1%) | 1 (ref) |  |  |  |
| 30-34 | 13,248 (29.1%) | 1.04 | (1.01, 1.06) |  |  |
| ≥35 | 5,431 (11.9%) | 1.05 | (1.02, 1.08) | <0.001 | **<0.001**** |
| **Birth-related factors** | |  |  |  |  |
| **Maternal infection during pregnancy** |  |  |  |  |  |
| No | 43,628 (96%) | 1 (ref) |  |  |  |
| Yes | 1,830 (4%) | 0.96 | (0.92, 1.01) | 0.14 | 0.30 |
| **Paternal infection during pregnancy** |  |  |  |  |  |
| No | 45,069 (99.1%) | 1 (ref) |  |  |  |
| Yes | 389 (0.9%) | 1.03 | (0.93, 1.15) | 0.53 | 0.61 |
| **Maternal genitourinary tract infection during pregnancy** |  |  |  |  |  |
| No | 44,951 (98.9%) | 1 (ref) |  |  |  |
| Yes | 507 (1.1%) | 0.85 | (0.78, 0.94) | <0.001 | **<0.05*** |
| **Congenital malformation of the mouth or digestive system** |  |  |  |  |  |
| No | 45258 (99.6%) | 1 (ref) |  |  |  |
| Yes | 200 (0.4%) | 0.99 | (0.86, 1.15) | 0.91 | 0.95 |
| **C-section** |  |  |  |  |  |
| No | 40,652 (89.4%) | 1 (ref) |  |  |  |
| Yes | 4,806 (10.6%) | 1.01 | (0.98, 1.05) | 0.40 | 0.52 |
| **Gestational age** |  |  |  |  |  |
| <35 weeks | 698 (1.5%) | 1.02 | (0.95, 1.11) |  |  |
| 35-36 weeks | 1,323 (2.9%) | 0.96 | (0.91, 1.02) |  |  |
| 37-41 weeks | 39,497 (86.9%) | 1 (ref) |  |  |  |
| ≥42 weeks | 3,940 (8.7%) | 0.96 | (0.93, 0.99) | 0.07 | 0.18 |
| **Birth weight** |  |  |  |  |  |
| <2000 g | 516 (1.1%) | 1.03 | (0.94, 1.13) |  |  |
| 2000-2499 g | 1,055 (2.3%) | 0.97 | (0.91, 1.04) |  |  |
| 2500-3999 g | 35,596 (78.3%) | 1 (ref) |  |  |  |
| ≥4000 g | 8,172 (18%) | 1.017 | (0.99, 1.04) | 0.39 | 0.52 |
| Missing | 119 (0.3%) |  |  |  |  |
| **Somatic factors** |  |  |  |  |  |
| **Childhood infection** |  |  |  |  |  |
| No | 34,076 (75%) | 1 (ref) |  |  |  |
| Yes | 11,382 (25%) | 1.01 | (0.99, 1.03) | 0.40 | 0.52 |
| **Childhood bacterial infection** |  |  |  |  |  |
| No | 42,102 (92.6%) | 1 (ref) |  |  |  |
| Yes | 3,356 (7.4%) | 1.03 | (0.989, 1.065) | 0.16 | 0.31 |
| **Childhood viral infection** |  |  |  |  |  |
| No | 40,075 (88.2%) | 1 (ref) |  |  |  |
| Yes | 5,383 (11.8%) | 0.99 | (0.965, 1.024) | 0.68 | 0.75 |
| **Childhood other infection** |  |  |  |  |  |
| No | 38,874 (85.5%) | 1 (ref) |  |  |  |
| Yes | 6,584 (14.5%) | 1.01 | (0.986, 1.041) | 0.35 | 0.52 |
| **Psychosocial factors** | |  |  |  |  |
| **Foster care/Out-of-home care** |  |  |  |  |  |
| No | 45,052 (99.1%) | 1 (ref) |  |  |  |
| Yes | 406 (0.9%) | 0.997 | (0.90, 1.11) | 0.95 | 0.95 |
| **In-home care** |  |  |  |  |  |
| No | 45,051 (99.1%) | 1 (ref) |  |  |  |
| Yes | 407 (0.9%) | 1.04 | (0.94, 1.15) | 0.46 | 0.56 |
| **Paternal education** |  |  |  |  |  |
| Elementary school | 10,066 (22.1%) | 1 (ref) |  |  |  |
| High school or vocational school | 21.406 (47.1%) | 1.02 | (0.99, 1.04) |  |  |
| Academic degree | 12,264 (27%) | 1.09 | (1.06, 1.12) | <0.001 | **<0.001**** |
| Missing | 1,722 (3.8%) |  |  |  |  |
| **Maternal education** |  |  |  |  |  |
| Elementary school | 11,656 (25.6%) | 1 (ref) |  |  |  |
| High school or vocational school | 18,503 (40.7%) | 1.022 | (0.997, 1.047) |  |  |
| Academic degree | 14,234 (31.3%) | 1.102 | (1.074, 1.131) | <0.001 | **<0.001**** |
| Missing | 1,065 (2.3%) |  |  |  |  |
| **Paternal income** |  |  |  |  |  |
| 1st quintile | 6,268 (13.8%) | 1 (ref) |  |  |  |
| 2nd quintile | 8,412 (18.5%) | 0.99 | (0.95, 1.02) |  |  |
| 3rd quintile | 9,447 (20.8%) | 0.97 | (0.94, 1.01) |  |  |
| 4th quintile | 10,079 (22.2%) | 0.99 | (0.96, 1.03) |  |  |
| 5th quintile | 10,886 (23.9%) | 1.04 | (1.01, 1.08) | <0.001 | **<0.001**** |
| Missing | 366 (0.8%) |  |  |  |  |
| **Maternal income** |  |  |  |  |  |
| 1st quintile | 6150 (13.5%) | 1 (ref) |  |  |  |
| 2nd quintile | 9576 (21.1%) | 0.954 | (0.923, 0.987) |  |  |
| 3rd quintile | 10323 (22.7%) | 0.937 | (0.906, 0.969) |  |  |
| 4th quintile | 9797 (21.6%) | 0.979 | (0.947, 1.013) |  |  |
| 5th quintile | 9472 (20.8%) | 0.993 | (0.96, 1.027) | <0.001 | **<0.001**** |
| Missing | 140 (0.3%) |  |  |  |  |
| **Parental psychiatric history** |  |  |  |  |  |
| No diagnosis | 39,518 (86.9%) | 1 (ref) |  |  |  |
| Other psychiatric diagnosis | 5,774(12.7%) | 1.01 | (0.98, 1.04) |  |  |
| Eating disorder diagnosis | 166 (0.4 %) | 1.20 | (1.02, 1.40) | 0.08 | 0.17 |

*Note.*  Multinomial logistic regressions were conducted on the sub-cohort with the risk factors as outcomes and the polygenic risk score for anorexia nervosa as the exposure to estimate the association between the levels of the risk factors and the anorexia nervosa polygenic risk score. *= significant at the p < 0.05 level. **= significant at the p < 0.001 level. *Abbreviations:* N=sample size; OR=odds ratio; CI=confidence interval. P-values were adjusted using the Benjamini-Hochberg correction table-wise within the association analyses.
